# Unproductive alternative splicing and nonsense mRNAs: A widespread phenomenon among plant circadian clock genes

**DOI:** 10.1186/1745-6150-7-20

**Published:** 2012-07-02

**Authors:** Sergei A Filichkin, Todd C Mockler

**Affiliations:** 1 Department of Botany and Plant Pathology and Center for Genome Research and Biocomputing, Oregon State University, Corvallis, OR, 97331, USA; 2 Donald Danforth Plant Science Center, St. Louis, MO, 63132, USA; 3 Division of Biology and Biomedical Sciences, Washington University, St. Louis, MO, 63110, USA

**Keywords:** Arabidopsis thaliana, Alternative splicing, Circadian clock, RNA-seq, Intron retention, Cassette exon, Nonsense mRNAs, Premature termination codon, CIRCADIAN CLOCK ASSOCIATED 1 (CCA1), LATE ELONGATED HYPOCOTYL (LHY), REVEILLE 2 (RVE2).

## Abstract

**Background:**

Recent mapping of eukaryotic transcriptomes and spliceomes using massively parallel RNA sequencing (RNA-seq) has revealed that the extent of alternative splicing has been considerably underestimated. Evidence also suggests that many pre-mRNAs undergo unproductive alternative splicing resulting in incorporation of in-frame premature termination codons (PTCs). The destinies and potential functions of the PTC-harboring mRNAs remain poorly understood. Unproductive alternative splicing in circadian clock genes presents a special case study because the daily oscillations of protein expression levels require rapid and steep adjustments in mRNA levels.

**Results:**

We conducted a systematic survey of alternative splicing of plant circadian clock genes using RNA-seq and found that many *Arabidopsis thaliana* circadian clock-associated genes are alternatively spliced. Results were confirmed using reverse transcription polymerase chain reaction (RT-PCR), quantitative RT-PCR (qRT-PCR), and/or Sanger sequencing. Intron retention events were frequently observed in mRNAs of the CCA1/LHY-like subfamily of MYB transcription factors. In contrast, the *REVEILLE2* (*RVE2*) transcript was alternatively spliced via inclusion of a "poison cassette exon" (PCE). The PCE type events introducing in-frame PTCs are conserved in some mammalian and plant serine/arginine-rich splicing factors. For some circadian genes such as *CCA1* the ratio of the productive isoform (i.e., a representative splice variant encoding the full-length protein) to its PTC counterpart shifted sharply under specific environmental stress conditions.

**Conclusions:**

Our results demonstrate that unproductive alternative splicing is a widespread phenomenon among plant circadian clock genes that frequently generates mRNA isoforms harboring in-frame PTCs. Because LHY and CCA1 are core components of the plant central circadian oscillator, the conservation of alternatively spliced variants between CCA1 and LHY and for *CCA1* across phyla [2] indicates a potential role of nonsense transcripts in regulation of circadian rhythms. Most of the alternatively spliced isoforms harbor in-frame PTCs that arise from full or partial intron retention events. However, a PTC in the *RVE2* transcript is introduced through a PCE event. The conservation of AS events and modulation of the relative abundance of nonsense isoforms by environmental and diurnal conditions suggests possible regulatory roles for these alternatively spliced transcripts in circadian clock function. The temperature-dependent expression of the PTC transcripts among members of *CCA1/LHY* subfamily indicates that alternative splicing may be involved in regulation of the clock temperature compensation mechanism.

**Reviewers:**

This article was reviewed by Dr. Eugene Koonin, Dr. Chungoo Park (nominated by Dr. Kateryna Makova), and Dr. Marcelo Yanovsky (nominated by Dr. Valerian Dolja).

## Background

Interrogation of the transcriptomes of several plant species using genome-scale expression microarrays revealed that many plant genes display diurnally driven rhythmic daily changes in mRNA abundance [1]. The rhythmic expression of the majority of the core clock and circadian associated genes is phased to remarkably similar times of day in *Arabidopsis thalianaPopulus* (poplar), *Brachypodium*, and *Oryza* (rice). Furthermore, the core circadian clock genes of *Oryza* subspecies *japonica* and *indica* displayed nearly indistinguishable oscillation profiles and phase calls [1]. The microarray platform has been a method of choice for genome-scale profiling of daily cyclical changes in mRNA abundance. However, expression microarrays have a substantial limitation: in most cases probe sets cannot differentiate among alternatively spliced isoforms of an mRNA. Therefore, for many alternatively spliced genes the overall expression values represent an average of signals generated by the mixture of mRNA variants. In contrast, the massively parallel RNA sequencing (RNA-seq) method is a high-resolution alternative to the expression arrays. RNA-seq coupled with bioinformatic analyses allows direct inference of transcript structures relative to the reference genome and unbiased characterization of alternatively spliced events.

Our recent RNA-seq survey of the *Arabidopsis* transcriptome suggested that some circadian clock genes undergo extensive alternative splicing (AS) yielding single or multiple splice isoforms [2]. Further, some specific splicing events were conserved across distantly related species of mono- and dicotyledonous plants [2]. For example, RNA-seq data predicted that alternatively spliced mRNA of the *CIRCADIAN CLOCK ASSOCIATED 1 (CCA1*) gene retains intron 4 (I4R) resulting in the introduction of several in-frame premature termination codons (PTCs). Validation of the I4R event in *CCA1* gene using an isoform-specific set of primers and quantitative reverse transcription polymerase chain reaction (qRT-PCR) showed that nonsense I4R *CCA1* transcripts can accumulate at high levels relative to the productive isoform in response to certain environmental stimuli such as high-intensity light stress [2]. Furthermore, despite substantial differences in nucleotide sequence, intron length, and the context of splice signals in homologs, the I4R event in *CCA1* mRNA was highly conserved among *CCA1* clock genes in both eudicots (*A. thaliana* and *P. trichocarpa*) and monocots (*O. sativa* and *B. distachyon*) [2]. This notable conservation suggests that the some AS events in circadian genes are highly conserved and therefore may play specific roles in the functioning of the plant circadian clock, possibly through the regulation of the abundance of circadian clock mRNAs. Here, we conducted a further survey and characterization of the AS events in plant circadian genes. Collectively, the RNA-seq, RT-PCR, qRT-PCR, and/or Sanger sequencing data suggested that several plant circadian genes of the central oscillator are extensively alternatively spliced.

## Results

### Strategy for discovery and identification of novel alternative splicing events in circadian genes

**Figure 1:**
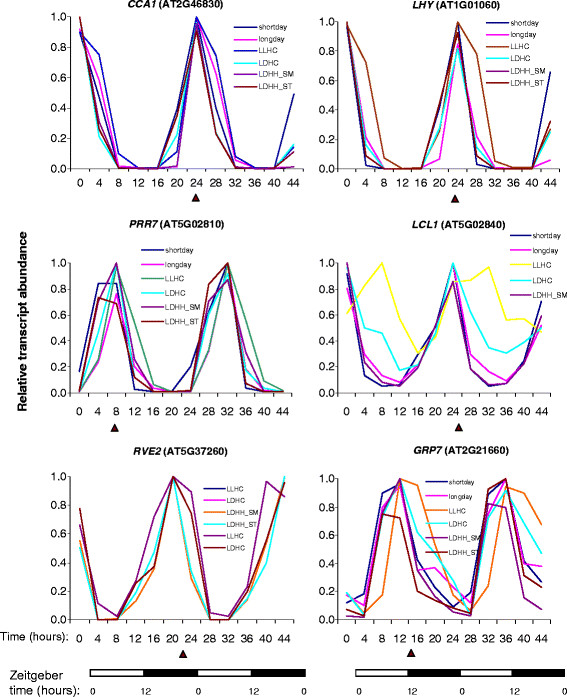
**Examples of diurnal expression patterns of some*Arabidopsis*circadian genes surveyed for alternative splicing events.** The time points when tissues were collected for RNA isolation are indicated by triangles. Gene expression patterns under different environmental conditions were obtained using the DIURNAL portal and microarray database. Detailed descriptions of diurnal conditions and experimental set up are available at http://diurnal.cgrb.oregonstate.edu/.

**Figure 2:**
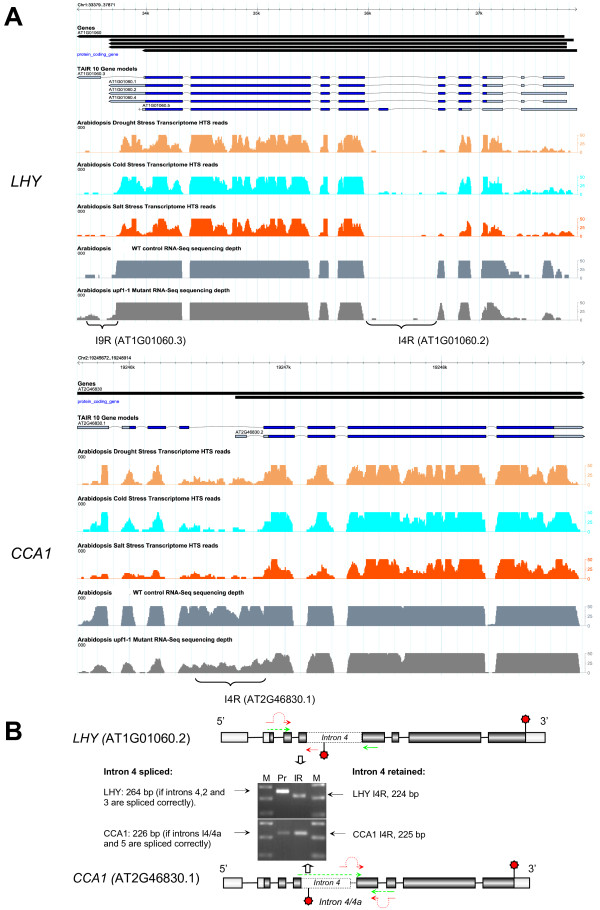
**Examples of the discovery and characterization of alternative splicing events in circadian genes and identification and validation of the I4R events in*LHY*and*CCA1*.** (**A**) Potential alternative splicing events were suggested by the distribution of RNA-seq reads density along gene features. The screen shots of the *CCA1* and *LHY* loci are from the *Arabidopsis* RNA-seq GBrowse. Histograms represent the density of coverage of gene features by Illumina reads (GBrowse tracks of HTS reads) under different abiotic stress conditions (e.g., salt, drought, and cold treatments). The tracks "WT control" and "upf1-1" represent read density in the wild-type control and in the *upf1-1* NMD mutant, respectively. The fourth introns in both the *CCA1* and *LHY* transcripts display an increase in read coverage (bracketed) under specific treatments suggesting that these introns may be retained more often under certain conditions. The I9R event in *LHY* is also represented by an increase in read coverage on the 3' UTR distal portion (bracketed) in the *upf1-1* NMD mutant. The numbers of *LHY* introns 4 and 9 correspond to the TAIR10 gene models AT1G01060.2 and AT1G01060.3, respectively. The *CCA1* intron 4 corresponds to AT2G46830.1. All three IR events were confirmed by RT-PCR and qRT-PCR (Figures 2, 3 and 5, Table 1). The scale of the read depth histogram is limited to a maximum of 50 reads. (**B**) Primer design strategy developed for validation of the I4R events in *CCA1* and *LHY* transcripts. Dark boxes depict protein-coding exons. The exons in the 5' and 3' UTRs are indicated by light boxes. The oligonucleotide primers used for the amplification of the constitutive and alternative splicing events are indicated by green and red arrows, respectively. The I4R events are depicted by dashed boxes. A small nested intron 4a in *CCA1* is indicated by a dashed line. Normal and premature termination codons are shown by top and bottom star symbols, respectively. The expected sizes of RT-PCR products are given in base pairs (bp). The gene models are not drawn to scale. The inset in (B) shows the RT-PCR confirmation of the *LHY* and *CCA1* transcripts with spliced and retained intron 4. Expected RT-PCR product sizes for the *LHY* transcript when intron 4 is spliced correctly is 264 bp; the size of the I4R isoform (if intron 4 is retained and introns 2 and 3 spliced out) is 224 bp. For *CCA1* the predicted product sizes are 226 bp (introns 4/4a and 5 spliced out) and 225 bp for the I4R isoform (introns 4a and 5 are spliced out but intron 4 is not). RT-PCR products were separated on a 2 % agarose gels and stained with ethidium bromide. ‘M’ - molecular size markers (markers correspond from bottom to top to 100, 200, and 300 bp).

To explore the extent and conservation of alternative splicing (AS) among plant clock genes we evaluated previously obtained *Arabidopsis* RNA-seq datasets [2]. Oscillating expression patterns for these genes under different diurnal conditions observed using microarray experiments are shown in Figure 1. All of the selected genes cycled under at least one diurnal condition with peak expression at different times of day. To maximize the sensitivity for minor spliced products and to avoid a possible light/dark-dependent shift in isoform ratios, the sampling was done at the phase of peak expression for each tested genes as shown in Figure 1. The distributions of RNA-seq reads plotted across gene features for the majority of known *Arabidopsis* circadian genes were first visually inspected in GBrowse for evidence of novel alternative splicing events as described in the Methods with emphasis on isoform-specific "diagnostic" gene regions that frequently display differential coverage by Illumina reads in response to specific stress treatments [2]. In addition, the distributions of the RNA-seq reads across introns annotated in The *Arabidopsis* Information Resource database (TAIR10; http://www.arabidopsis.org) were evaluated using the GENE-Counter package [3]. Twenty-four putative alternative splicing events in circadian clock-associated transcripts were selected for further analysis. Candidate AS events were validated using RT-PCR and/or qRT-PCR with oligonucleotide primers designed to specifically discriminate AS events. Selected RT-PCR products were additionally subjected to standard Sanger sequencing. Figure 2 depicts the strategy for identification and validation of predicted alternative splicing events in the *LHY* and *CCA1* transcripts. Notably, both genes showed conservation of the I4R event in spite of variation in length and limited similarity (data not shown) in intron 4 sequence.

### Pre-mRNAs of the CCA1/LHY-like subfamily of MYB transcription factors undergo extensive and conserved alternative splicing

**Figure 3:**
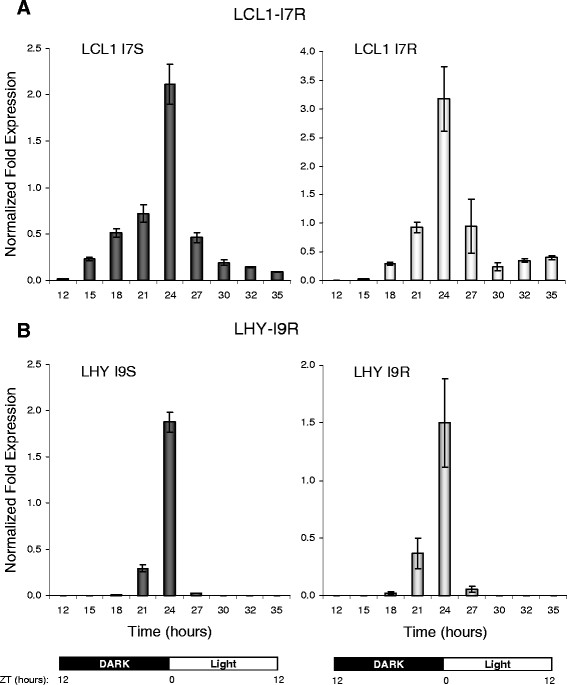
**Oscillating profiles of IR events in*LHY*and*LCL1*are regulated by photoperiodic conditions with expression peaks at dark/light transitions under constant temperature (20 °C).***LHY-I9S* and *LHY-I9R* transcripts correspond to those with spliced and retained intron 9 events, respectively. *LCL1-I7S* and *LCL1-I7R* are transcripts with spliced and retained intron 7 events, respectively. The sampling and diurnal conditions are described in Methods. The normalized fold change in expression was calculated using a –ΔΔCt method and the CFX Manager software as described in Methods.

The alternative splicing events in *Arabidopsis* circadian genes predicted from RNA-seq data are listed in Table 1. All of the genes listed have been implicated directly or indirectly in the function of the core circadian clock or are circadian-associated genes regulating clock output (reviewed in [4]). Using RT-PCR or qRT-PCR with alternative splicing event-specific primers and in some cases Sanger sequencing of cloned cDNAs and RT-PCR products, we confirmed that twelve *Arabidopsis* core clock or circadian clock-associated genes are alternatively spliced. Consistent with the earlier finding that ~55 % of alternatively spliced transcripts in *Arabidopsis* involve intron retention (IR) events [5], we found that most of the AS events in circadian genes result in either full or partial IR with alternative donor splice sites (Table 1). Furthermore, analysis of the sequences of the resulting splicing variants confirmed our previous observation [2] that most of the investigated AS events resulted in introduction of at least one in-frame PTC (Figure 2 and data not shown). Among alternatively spliced genes of the central circadian oscillator, *CCA1* and *LHY* represent the core feedback loop whereas *PRR7* and *PRR9* play roles in the morning loop [4]. Other central clock genes with IR events included the genes encoding REVEILLE (RVE) 7 and 8, homologs that belong to a CCA1/LHY-like subfamily of MYB transcription factors [6], which bind to the Evening Elements (EE) in the promoters of their target genes [7] and share some functional redundancy with CCA1 and LHY. Our results suggest that the pre-mRNAs of the core clock CCA1/LHY-like subfamily of MYB transcription factors undergo extensive alternatively splicing, predominantly involving IR events (Figures 2 and 3, Table 1).

**Table 1:** **Validation of predicted alternative splicing events in*Arabidopsis*circadian genes**

**Gene**	**AGI locus**	**Predicted AS event**	**Fragment size, bp**	**Confirmed by**
*LHY*	AT1G01060.2	Retained intron 4 (AT1G01060.2)	224	RT-PCR, qRT-PCR, Sanger sequencing
	AT1G01060.3	Retained intron 9 (AT1G01060.3)	188	RT-PCR
*LCL1*	AT5G02840.1	Retained intron 7	230	RT-PCR
	AT5G02840.1	Retained intron 8	135	RT-PCR
	AT5G02840.3			
*CCA1*	AT2G46830.1	Retained intron 4	224	RT-PCR, qRT-PCR, Sanger sequencing
*PRR7*	AT5G02810	Retained intron 3	208	RT-PCR, Sanger sequencing
*PRR9*	AT2G46790	Retained intron 3	172	RT-PCR
*CIR1, RVE2*	AT5G37260	Alternative Poison Cassette Exon (PCE) in intron 1	218	RT-PCR, Sanger sequencing
*CIR1, RVE2*	AT5G37260	Retained intron 3	225	RT-PCR, Sanger sequencing
*CKB3*	AT3G60250	Retained intron 3	153	RT-PCR
		Retained intron 4	272	RT-PCR
*ASG4*	AT1G01520	Retained intron 6	345	RT-PCR
*RVE7, EPR1*	AT1G18330	Retained intron 1	213	RT-PCR
*RVE8, LCL5*	AT3G09600	Retained intron 7	390	RT-PCR
		Retained intron 8	589	RT-PCR
*CCR2, GRP7*	AT2G21660	Retained portion of the intron 1 (alternative donor splice site)	184	RT-PCR, qRT-PCR, Sanger sequencing
*CCR1, GRP8*	AT4G39260	Retained portion of the intron 1 (alternative donor splice site)	188	RT-PCR

PCR conditions and primers used for validation of each AS event are compiled in Additional file 2.

### Unproductively spliced circadian transcripts can harbor PTCs at various locations in the mRNA

The location of a PTC within an mRNA can influence recognition of the transcript by the nonsense mediated mRNA decay (NMD) pathway in both mammals and plants and therefore may determine its NMD-eliciting properties. The distribution of AS events observed suggested that alternatively spliced circadian transcripts can harbor both early and late classes of PTCs (Table 1 and data not shown). The positions of retained introns along the length of the transcripts varied from early in *RVE7* (I1R) to intermediate in *CKB3* (I4R) to late in *LCL1* (I7R) (Table 1). We monitored the relative levels of the *LCL1* (I7R) transcripts during *LCL1* peak expression using qRT-PCR. The isoforms with both retained (I7R) and spliced (I7S) intron 7 displayed similar temporal profiles with the peak expression at the dark/light transition period (ZT0, Figure 3A). In five out of twelve of the circadian genes inspected (*LHY*, *LCL1*, *RVE2*, *RVE8*, and *CKB3*) we identified at least two alternative splicing events (Table 1). The combinatorial nature of the alternative splicing events in these genes may yield transcripts that harbor both early and late PTCs.

### Alternative splicing of 3’ untranslated regions

As initially suggested by RNA-seq read data (Additional file 1: Figure S1) and further validated by RT-PCR (Table 1), *LHY*, *LCL1*, and *RVE8/LCL5* mRNAs had IR events in their 3’ untranslated regions (3’ UTRs). A time course of *LHY* transcript accumulation showed that isoforms with a retained intron in the 3’ UTR (I9R) and the fully spliced form (I9S) peaked at the dark/light transition period (ZT0, Figure 3B). The retention of introns in the 3’ UTRs of *LCL1* (I8R), *LHY* (I9R), and *RVE8* (I8R) results in transcript variants with abnormally long (500–800 nucleotides) 3’ UTRs that may be detected and degraded by NMD. Indeed, RNA-seq read coverage of the *LHY* 3' UTR also suggested that the levels of the *LHY* I9R isoform are increased in the NMD-impaired *upf1-1* mutant (Additional file 1: Figure S1A).

### An early PTC is introduced in the RVE2 pre-mRNA via a cassette exon

**Figure 4:**
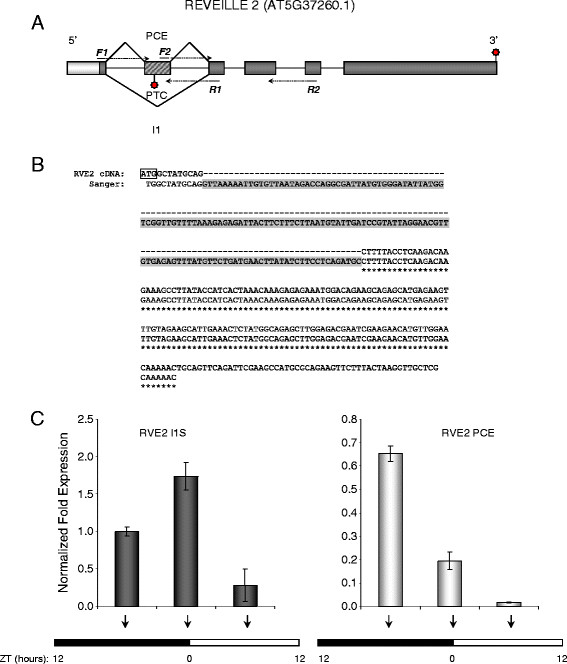
**Alternative splicing of*RVE2*pre-mRNA introduces an in-frame nonsense codon via a PCE event.** (**A**) The schematic representation of the AS event in intron 1 introducing a PCE in the *RVE2* transcript. The 5' UTR is shown by a light box, protein coding exons and the PCE are indicated by dark and hatched boxes, respectively. Binding sites for primers F1-R1 and F2-R2 used in RT-PCR are shown by arrows. Normal and premature termination codons are shown by the top and bottom stars. Dashed lines indicate primer portions spanning splice junctions. The gene model is not drawn to scale. (**B**) The alignment of the 5’ portion of *RVE2* cDNA (top) and Sanger sequences of the PCR products (bottom). The PCE sequence is highlighted in grey. The initiation codon is boxed. (**C**) Quantification of alternatively spliced *RVE2* mRNA harboring PCE event using qRT-PCR. The diurnal conditions and the sampling scheme are described in detail in Methods. Arrows indicate the sampling time points (ZT, hours). The relative transcript quantities were calculated using the –ΔΔCt method and CFX Manager software (BioRad). *GOG* mRNA was used as an internal reference. Vertical bars represent the standard error of the mean.

The RNA-seq data indicated a possible partial retention event in the first intron of the *RVE2* gene. Closer examination of the nucleotide sequence and cDNA evidence suggested that this alternative splicing of the RVE2 pre-mRNA likely generates a poison cassette exon (PCE) – an incorporation of a short alternative exon that harbors an early in-frame PTC [8]. Analysis of the spliced junction sequences flanking this PCE by RT-PCR and Sanger sequencing confirmed the presence of the predicted splice junctions in the RVE2 mRNA (Figure 4). The occurrence of the PCE event in *RVE2* was corroborated by two full-length cDNAs (TAIR accessions BT010947 and BX834498). Neither of these two cDNAs contained an open reading frame starting that began with a canonical AUG initiation codon in close proximity to their 5’ termini (data not shown). Thus, the efficient translation of the truncated protein from *RVE2* mRNAs harboring a very early PTC is unlikely. Further evaluation of the PCE event in *RVE2* by qRT-PCR showed that under normal physiological conditions the PCE-carrying *RVE2* transcripts accumulated to substantial levels (Figure 4). Conserved PCE-type AS events have been described in several mammalian [8] and *Arabidopsis*[2] serine/arginine-rich (SR) splicing factors. However, to our knowledge this is the first example of PCE occurrence in a plant circadian gene.

### Alternative splicing of circadian clock-output genes

Interrogation of AS events using primers designed to hybridize to expected splice junctions confirmed that transcripts of *CIRCADIAN RHYTHM AND RNA BINDING2* (*CCR2*, also known as *GLYCINE RICH PROTEIN7* or *GRP7*) and *GLYCINE RICH PROTEIN 8* (*CCR1* or *GRP8*) are also alternatively spliced as suggested by previous studies [9-11]. Validation of RNA-seq data using RT-PCR and primers to splice junction regions showed that some *GRP7* transcripts retained a portion of the first intron through the selection of an alternative donor splice site (Table 1 and Additional file 2). We confirmed that a similar partial retention event also occurred in the first intron of the GRP8 transcript through use of an alternative donor splice site. These two analogous events led to incorporation of early PTCs in both GRP7 and GRP8 transcripts (data not shown) and offer another example of conservation of specific AS events among homologs with overlapping but distinct functions.

### Nonsense transcript isoforms can accumulate to substantial levels depending on environmental conditions

**Figure 5:**
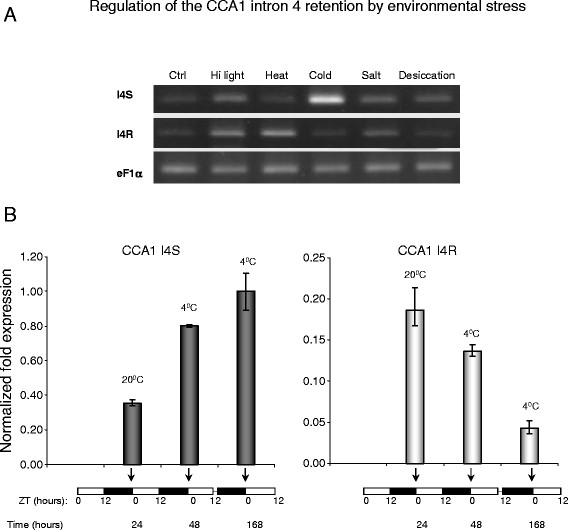
**Regulation of the expression levels of alternatively spliced*CCA1*isoforms by environmental stress.** (**A**) Semi-quantitative RT-PCR analysis of the I4R event in *CCA1* transcripts under different abiotic stress treatments. The relative abundance of the PTC-harboring *CCA1* I4R isoform changes compared to the full-length spliced variant (I4S) after cold stress treatment. Two-week-old seedlings were treated as previously described [2]. Ctrl - untreated seedlings, Hi light - high intensity light, Heat - heat stress (42 °C), Cold - cold stress (4 °C), Salt - high salinity (0.5 M sodium chloride), Desiccation - dehydration (polyethylene glycol treatment). *eF1α* mRNA was used to demonstrate an equal PCR amplification of cDNAs. PCR was carried out for 16 cycles for all the reactions. PCR products were separated in 2 % agarose gels and stained by ethidium bromide. (**B**) qRT-PCR analysis of cold stress-induced changes in relative abundance of the *CCA1* splice variants. The normalized expression of the *CCA1* transcripts with spliced intron 4 (CCA1 I4S) increased after 12 and 168 hours of the cold treatment (4 °C). In contrast, the normalized fold expression of the I4R transcripts sharply decreased following the treatment at 4 °C. *GOG* was used as a reference housekeeping transcript. The sampling and conditions of the time course are described in detail in the Methods. The normalized fold change of expression of the target transcripts was calculated using a –ΔΔCt method and CFX Manager software. Vertical bars denote the standard error of the mean. Both RT-PCR and qRT-PCR were performed using splicing event-specific oligonucleotide primers (see Additional file 2). ZT - Zeitgeber time (hours).

Analysis of several circadian genes by quantitative RT-PCR (qRT-PCR) showed that alternatively spliced nonsense transcripts of several genes accumulated to moderate to high levels relative to the full-length protein-encoding counterparts. For example, depending on the stress treatment, the ratio of *CCA1* full-length and PTC-containing isoforms shifted drastically up or down (Figure 5A). Heat stress treatment decreased the full-length isoform to marginal levels accompanied by a sharp increase in the PTC-containing transcript, suggesting that a dynamic balance between these isoforms is regulated by temperature. Further, over the time course, the PTC-containing *CCA1* transcript levels decreased 24 hours after cold stress treatment and remained low as long as plants were maintained in the cold (Figure 5B). The normalized expression of the full-length transcript with a correctly spliced intron 4 increased and remained at a high level over the duration of cold treatment.

### Differential RNA-seq coverage of introns suggests additional intron-retention events in key circadian genes

To identify additional intron retention events in circadian genes we subjected *Arabidopsis* seedlings to heat stress treatments followed by RNA-seq analysis as previously described [2]. The RNA-seq dataset derived from the heat-treated plants was compared to the data from untreated controls. The statistical significance of differential expression of the introns of TAIR-annotated genes was evaluated using the GENE-Counter package [3] as described in the Methods. The differential coverage of introns by the 40-mer Illumina reads before and after heat stress treatment suggested that in addition to already validated transcripts (Table 1) additional IR events may occur in other circadian clock genes; these genes are listed in Additional file 3. The most notable of these potentially alternatively splice genes are the key circadian genes *TIME FOR COFFEE* (*TIC*), *PHYTOCLOCK 1* (*PCL1*), *and LOV KELCH PROTEIN 2* (*LKP2*)*.* Therefore, the repertoire of unproductive alternative splicing events in circadian clock genes is likely to be even more diverse than those validated here. The relative levels of these putative novel isoforms with retained introns are likely to change in response to the thermal stress.

## Discussion

Our survey of the *Arabidopsis thaliana* transcriptome revealed that at least twelve genes associated with the circadian clock are subject to extensive alternative splicing. Three circadian genes harbored more than one AS event, suggesting a complex combinatorial pattern of resulting transcript isoforms. Notably, several AS events were conserved between homologous genes with overlapping but distinct functions. The specific alternative splicing events in some of the core clock genes such as *CCA1* are conserved across distantly related plant species [2]. Both CCA1 and LHY are morning-expressed transcription factors that act as negative regulators of TIMING OF CAB EXPRESSION 1 (TOC1) in the core oscillator feedback loop (reviewed in [4]). CCA1 and LHY repress TOC1 by binding to the EE motif within its promoter [4,12,13]). In turn, evening-expressed TOC1 activates the expression of CCA1 and LHY. The conservation of the *CCA1* intron 4 retention event in different phyla suggests a selective evolutionary pressure to preserve analogous alternative splicing events in certain circadian genes [2]. In this study we demonstrated that *LHY*, a *CCA1* homolog with overlapping but distinct circadian clock functions, also was alternatively spliced through an analogous I4R event.

Further, IR events were observed in the 3’ UTRs of transcripts from *LHYLCL1*, and *RVE8* genes. Because 3’ UTRs longer than 300 nt can elicit an NMD response in *Arabidopsis*[14] it is likely that the *LHYLCL1*, and *RVE8* transcripts harboring IR events in their 3' UTRs are targeted by NMD. Indeed, the distribution of RNA-seq read coverage of the 3' UTRs in the NMD-impaired mutant *upf1-1* indicated that the *LHY-I9R* isoform was present at higher levels than those observed in the wild-type strain, and it is therefore likely that this isoform is degraded through the NMD pathway.

The *GRP7*/*GRP8* pair of homologs further support the notion that that similar AS events introducing in-frame PTCs are conserved among homologous but functionally non-redundant circadian genes. The circadian slave oscillator GRP7 modulates clock output networks [4,10], regulates mRNA export from the nucleus to the cytoplasm during cold stress [9], and mediates pathogen defense responses [15]. GRP7 auto-regulates its abundance via a negative post-transcriptional feedback loop; it binds to its own pre-mRNA and influences splice site selection [9,10]. Recently, it was proposed that the reciprocal regulation of GRP7 and GRP8 occurs via an interlocked feedback loop [11]. Moreover, the alternative splicing of *GRP7* and *GRP8* pre-mRNAs influences circadian oscillations of both transcripts and could be coupled to the NMD pathway. The examples described above suggest certain unproductive AS events in circadian genes have been under stabilizing selection, and therefore it is likely that these transcripts have roles in regulation of the circadian clock.

The functional significance of *CCA1* and *LHY* nonsense transcripts is not known. It is possible that PTC-harboring *CCA1* and *LHY* mRNAs are rapidly degraded by NMD machinery. Therefore, the PTC-containing isoforms of *CCA1* and *LHY* may contribute to regulation of the core feedback loop by fine-tuning the abundance of the full-length protein-coding transcripts. Another possibility is that transcripts harboring early PTCs in the 5’ region of the mRNA escape NMD detection and enter the steady-state translation pool; this occurs in mammalian cells [16]. In this scenario truncated CCA1 and LHY proteins carrying DNA binding MYB domains may compete with the full-length proteins for binding to *cis* regulatory sites. Because CCA1 and LHY form homo- and heterodimers *in vivo*[17], truncated proteins may also disrupt the assembly of functional dimers and may act as dominant negative regulators.

We demonstrate that alternative splicing via poison cassette exon leads to incorporation of early PTCs in *RVE2* transcripts. Conserved PCE events have been described in the mRNAs of several human SR splicing factors [8]. The introduction of PTCs in mRNAs through PCE events was proposed to play a role in homeostatic regulation of abundance of the productive (i.e., encoding a full-length protein) transcript [8]. Previously, we characterized an analogous PCE event in the mRNA of the *Arabidopsis* splicing factor SR34 [2]. However, to our knowledge, the PTC-containing isoform of *RVE2* mRNA is the first example of a characterized PCE event that introduces a nonsense codon into the mRNA of a key plant circadian clock gene. This finding suggests that the PCE-type AS events that occur in mammalian SR splicing factors genes [8] may represent a widespread strategy for introducing in-frame PTCs that is common among eukaryotic organisms including plants.

Cellular localization, recognition by NMD machinery, and translational activity of nonsense circadian transcripts remain unknown. According to the “stochastic noise” hypothesis many unproductive AS events may represent a "cellular noise" of an inherently error prone splicing machinery [18]. However, the “noise” hypothesis cannot explain the fact that unproductive AS is widespread among eukaryotes: Up to 10 % of all mRNAs in yeast, fly, and human cells harbor PTCs [19]. A “noisy” hypothesis is also in disagreement with the notion that many alternative splicing events (including those described here for several circadian genes) are highly conserved between homologs and/or across distantly related species [2,8].

The final ratio of transcript isoforms is dictated by the choice of splicing signals and is determined by the selection and concentration of specific splicing factors with antagonistic or overlapping functions. The result of an accumulation of alternatively spliced transcripts could be a functional down regulation of the expression of a gene under specific conditions. Conserved alternative splicing patterns in plant SR splicing factors transcripts can be activated developmentally or by changes in external conditions such as environmental stresses [20-22]. This notion is in line with our finding that the level of the PTC-containing *CCA1* transcript sharply decreased in cold-treated plants suggesting that unproductive splicing may be involved in the clock temperature compensation mechanism. Indeed, because the levels of *CCA1* transcripts increased at lower temperatures, CCA1 was proposed to be responsible for low temperature compensation of the circadian clock [23].

Although the destiny and regulatory significance of alternatively spliced PTC-harboring transcripts in general and in circadian genes in particular remain unclear, the discovery of widespread unproductive AS in plant circadian genes raises a question of whether alternatively spliced mRNA serve as regulators of the circadian clock. It is possible that unproductively spliced mRNAs function compete with full-length mRNAs in a post-transcriptional mechanism for regulation of protein expression; these PTC-containing transcripts may eventually enter the NMD pathway.

Whether alternatively spliced isoforms can be translated normally or are recognized and targeted by NMD appears to depend on the location of the PTC within the mRNA. Indeed, recent evidence suggests that some transcripts with late PTCs (those in the 3’ region of the message) are translated to produce truncated proteins that act as dominant negative regulators. The examples of such regulation have been previously characterized biochemically for hnRNP A/B proteins [24] and SR splicing factors [25]. In plants, early PTCs can either escape NMD [26] or elicit an NMD response less efficiently than the PTCs located in the middle of the coding sequence [27]. We hypothesize that the transcripts harboring relatively early PTCs (such as *CCA1-I4R* and *LHY-I4R*) enter the translating pool and the resulting truncated proteins compete with and/or antagonize the normal functions of their full-length counterparts.

Increasing evidence suggests that the coupling of alternative splicing and NMD is a common phenomenon in mammalian cells and plays an important role in regulation of gene expression via regulated unproductive splicing and translation (RUST) [8,28]. According to the RUST hypothesis, the abundance of a full-length protein-encoding transcript can be regulated by shifting the pre-mRNA splicing towards PTC-containing isoforms that are degraded by the NMD pathway. Because RUST occurs at the post-transcriptional level, it may provide a rapid response to the cell's changing requirements for particular mRNAs. Several instances of modulating productive transcript levels via unproductive alternative splicing and a negative feedback loop have already been described. For example, the mammalian splicing factor SC35 self-regulates its expression by modulating levels of unproductive splicing of the 3’ UTR of its own pre-mRNA [29].

We propose that RUST regulation of the PTC-containing *Arabidopsis* core circadian gene transcripts could be of particular importance because it allows rapid post-transcriptional adjustments in abundance of the oscillating productive transcript isoforms over the course of day. This hypothesis is in line with the recent discovery that AS of the core clock gene *PRR9* is regulated by PROTEIN ARGININE METHYL TRANSFERASE 5 (PRMT5). Mutations in PRMT5 (which transfers methyl groups to arginine residues of certain spliceosomal proteins) impair circadian rhythms in *Arabidopsis* by causing severe changes in levels of *PRR9* splice isoforms [30]. PRMT5 expression is regulated by light/dark cycles, and it is a likely candidate for the factor that links environmental changes to levels of unproductive spliced products of plant circadian clock genes.

## Conclusions

Many plant circadian clock genes undergo extensive alternative splicing that impacts the sequence of mRNA in both coding and untranslated regions. Most but not all of the alternatively spliced isoforms arise from full or partial intron retention events. In different circadian genes, the unproductive alternative splicing can introduce early, intermediate, or late in-frame premature termination codons. The relatively high abundance of transcripts that contain premature termination codons and the conservation of several characterized splicing events in homologous proteins of *Arabidopsis* and in multiple species suggest that the alternative splicing may play a role in regulation of the cyclical daily expression of plant circadian genes.

## Methods

### Plant growth conditions and sampling scheme

Wild-type control and *upf1-1* mutant (ABRC stock CS870823, http://www.arabidopsis.org/) *Arabidopsis thaliana* (ecotype Columbia 0) seedlings were germinated and grown on plates containing Murashige and Skoog medium for two weeks. The seedlings were subjected to abiotic stress treatments such as high-intensity light, heat, cold, high salinity, and dehydration as previously described [2]. For quantitative RT-PCR analysis, four-week-old *Arabidopsis* plants were entrained for ten days at constant temperature (20 °C) in a PGR15 growth chamber (Conviron) with day/night cycles of 12 hours light and 12 hours dark and a light intensity of 250 μmol m^-2^ s^-1^. For the diurnal profiling of splice variants (as shown in Figure 3) leaf tissues were collected every 3 hours. For the cold treatments (as shown in Figure 5) plants were first entrained as described above. The first time point (ZT0, 24 hours, Figure 5) was collected at 20 °C. Then, the temperature was decreased to 4 °C during the nights. The second time point (48 hours) was collected after exposing plants to 4 °C for 12 hours in the dark. The final time point (168 hours) was collected after the plants were entrained for an additional 96 hours at 4 °C during the night.

### RNA isolation

Seedlings or leaf tissues were frozen in liquid nitrogen and ground to a fine powder using mortar and pestle. Total RNA was isolated using modification of previously described protocol [2]. Briefly, RNA was extracted using The Plant RNA Reagent (Invitrogen) and treated for 10 min at 65 °C with RNAsecure reagent (Ambion). To eliminate genomic DNA amplification, the RNA was treated for 15 min at 37 °C with RNase-free Turbo DNase (Ambion). Total RNA was additionally purified using RNAeasy Mini RNA kit (Qiagen) following the manufacturer’s RNA clean up protocol. Integrity and concentration of the RNA were monitored using Bioanalyzer 2100 (Agilent Technologies) and ND-1000 spectrophotometer (Thermo Fisher Scientific). Only RNA samples with RNA integrity numbers greater than 6 was used for cDNA synthesis.

### Identification of potential alternative splicing events

The cycling profiles of *Arabidopsis* circadian genes were downloaded using Diurnal Search Tool through the Diurnal portal (http://diurnal.cgrb.oregonstate.edu/). The gene models and RNA-seq coverage among the key circadian clock genes were visually inspected using *Arabidopsis* RNA-seq GBrowse (http://athal.cgrb.oregonstate.edu/cgi-bin/gbrowse/arabidopsis-gbrowse/). The nucleotide sequences of candidate genes were obtained from *Arabidopsis* TAIR10 annotation (http://www.arabidopsis.org/) and splice junction specific primers were designed as outlined below.

### Primer design and validation of isoform-specific AS events

A strategy for designing splice junction-specific primers was described previously [2]; examples are depicted in Figure 2. One of the primers was designed to hybridize to the splice junction of interest, the other to intron or exon sequence up- or downstream. The final optimization of primer pairs sequence was performed by using the Primer3 tool (http://primer3.sourceforge.net/). The sequences of oligonucleotide splice isoform-specific primers are listed in Additional file 2.

### cDNA synthesis and RT-PCR

The first cDNA strand was synthesized using 100 ng of total mRNA, an anchored oligo d(T) primer, and Superscript III reverse transcriptase using the Invitrogen first-strand cDNA synthesis kit according to the manufacturer’s protocol. The first-strand cDNA reaction was diluted 10-fold and approximately 50 ng of cDNA was used as PCR template. The PCR amplification was carried out using DNA Engine Dyad (Bio-Rad) instrument and Phusion polymerase (New England BioLabs) in the presence of 2 % (v/v) dimethylsulfoxide. The generic “touch down” PCR protocol included 7 “touch down” cycles for the increased specificity (98 °C for 10 sec, 67 °C for 30 sec, 72 °C for 1 min, with annealing temperature decreasing 2 °C at each consequent cycle) followed by 20 to 30 cycles of 95 °C for 15 sec, 57–60 °C for 30 sec, and 72 °C for 1 min and a final extension at 72 °C for 10 min. The number of amplification cycles and annealing temperatures were optimized for each target cDNA depending on transcript abundance and melting temperatures of primer/target duplexes, respectively. Detailed descriptions of individual PCR protocols used for each target are summarized in Additional file 2. The PCR products were separated in 2.5 % agarose gels and stained with ethidium bromide. *GOG* (AT5G11980) and *EF1α* (AT5G60390) were used as internal housekeeping mRNA references to ensure an equal RT-PCR amplification and gel loading. Contamination by genomic DNA was monitored by amplification of the genomic *ACTIN2* (AT3G18780) sequence.

### Quantitative RT-PCR

qRT-PCR was performed using a Bio-Rad CFX96 Real-Time instrument and QuantiTect SYBR Green PCR master mix (Qiagen). The amplification conditions for all qRT-PCR were: 95 °C for 15 sec; then 94 °C for 15 sec, 56 °C for 30 sec (at a slow ramp rate 2 °C per sec), 70 °C for 20 sec (plate read), 75 °C for 15 sec (plate read) for 40 cycles; followed by melting curve step of 65 °C to 95 °C in increments of 0.5 °C (plate read). The normalized fold expression was calculated using CFX Manager software (Bio-Rad) and the -ΔΔC_(t)_ method. *GOG* (AT5G11980) was used as a housekeeping reference gene for normalization of expression. The expected sizes of all qRT-PCR products were additionally confirmed by gel electrophoresis in 2 % agarose followed by staining with ethidium bromide. The sequences of oligonucleotide splice isoform-specific primers used in qRT-PCR are listed in Additional File 1.

### Sanger sequencing

Sanger sequencing was performed according to standard protocols using an ABI 3730 capillary sequencer (Applied Biosystems).

### Statistical analysis of the intron coverage by RNA-seq reads

RNA-seq datasets derived from the heat stress treated and untreated control seedlings. The statistical significance of differential expression of the TAIR-annotated introns was evaluated using GENE-Counter tool [3]. The introns were considered differentially expressed at the level of statistical significance if the P and Q values for normalized Illumina reads coverage were less or equal 0.05 (see Additional file 3). An additional filtering included for the fold change in Illumina read coverage (all the introns with fold coverage change less than two were removed from the list). Circadian genes were identified based on TAIR10 annotation (http://www.arabidopsis.org/).

## Abbreviations

A, Alternative splicing; Bp, Base pairs; cDNA, Complementary DNA; IR, Intron retention; NMD, Nonsense mediated mRNA decay; Nt, Nucleotides; PTC, Premature termination codon; PCE, Poison cassette exon; RNA-Seq, High throughput RNA sequencing; RT-PCR, Reverse transcription polymerase chain reaction, qRT-PCR, quantitative RT-PCR; RUST, Regulated unproductive splicing and translation; SR, Serine/arginine splicing factors; UTR, Untranslated region; ZT, Zeitgeber time.

## Competing interests

The authors declare that they have no competing interests.

## Authors’ contributions

SAF and TCM conceived the experimental design and wrote the paper. SAF developed RT-PCR and qRT-PCR validation assays for predicted splice isoforms, constructed RNA-seq libraries, conducted all other experiments including prediction of novel splice sites and evaluation of differential coverage of introns by Illumina reads. All authors read and approved the final manuscript.

## Reviewers’ Comments

Reviewer 1: Dr. Eugene V. Koonin

*Reviewer's comments:* Very interesting, solid work. The extensive alternative splicing yielding nonsense mRNAs of clock genes is demonstrated beyond doubt. The direct involvement of this phenomenon in clock regulation remains a hypothesis, and the authors are quite careful about. The only substantial question that I have is: would it be possible to compare the level of alternative splicing in clock genes to the overall background in Arabidopsis? Is it the case that the clock genes are particularly prone to alternative splicing in general and to accumulation of PTC in particular? An answer in the affirmative would extremely interesting and supportive of the regulation hypothesis but even a negative answer would be interesting.

*Authors' response:* Data from our previous transcriptome-wide RNA-seq study [REF [2] was incorporated into the Arabidopsis TAIR10 genome annotation and estimates that approximately 42 % of Arabidopsis genes are alternatively spliced. Currently, there are about 29 characterized core clock and circadian-associated genes in the TAIR10 annotation. Approximately half of them (15) are represented by two or more gene models. Therefore, at first glance the extent of alternative splicing in circadian genes appears similar to transcriptome-wide estimates. However, in this study we did not seek to explore statistics of alternative splicing among circadian genes *per se*. More importantly, we found that the patterns of alternative splicing in some key circadian genes can be very complex and in most cases generates nonsense isoforms. Moreover, circadian genes have a broad repertoire of alternative splicing including intron retention, alternative donor/acceptor sites, cassette exons, and alternatively spliced 5' and 3' UTRs. Thus, the central components of the clock oscillator *CCA1* and *LHY* are represented by two and five gene models, respectively. Interestingly, we found that only one intron 4 retention event (I4R) introducing PTCs downstream of their DNA binding domains is conserved between *CCA1* and *LHY* homologs. In addition, both the 5' and 3' UTRs of *LHY* are alternatively spliced suggesting that some of its isoforms may be regulated by NMD. Indeed, after this paper was submitted we found that the *LHY* isoform retaining an intron in its 3' UTR is up-regulated in NMD mutant (data not shown).

Concerning the question of whether the alternative splicing of the circadian genes generates more PTCs than average across the transcriptome, we have no statistically meaningful answer. However, the notion is that most of the alternative splicing events in circadian genes introduce PTCs and these events are likely to be at least as frequent as they are on average across the transcriptome. Not all PTCs will elicit mRNA degradation. Currently we are trying to determine which of the PTCs in circadian genes elicit NMD.

Reviewer 2: Dr. Chungoo Park

*Reviewer's comments:* In this manuscript, the authors showed that Arabidopsis thaliana circadian clock genes tend to be extensively alternatively spliced, and such isoforms especially retaining intron are subject to contain premature termination codons and to be conserved. However, these findings have been already observed by Filichkin et al. (2010) [Ref [2]. In this study, which is different from previous study?

*Authors' response:* Even though the results reported in this paper are broadly related to our previous studies they are clearly unique. Further, this study addresses a different set of fundamental biological questions related to the role of unproductive alternative splicing (UAS) in circadian clock regulation for the following reasons.

First, unlike our previous study, here we interrogated novel splice junctions (SJs) in a subset of circadian gene transcripts at specific times of day. To pinpoint the peak expression phase for each transcript we used our recently obtained diurnal microarray datasets [REF [2]. In contrast to the pooled sampling scheme this approach added an important advantage of enabling detection of minor splice variants at peak expression times, which can be missed if timing is not correct.

Second, using a diurnal time course sampling we showed that splice isoforms harboring in-frame premature termination codons (PTCs) can accumulate in a cyclical manner and in many (but not all) instances mirror the oscillations of the productive transcripts. Importantly, our time course data suggests a temporal shift in isoform ratios depending on the time of day or environmental conditions such as temperature changes.

Third, here we demonstrated that the conservation of similar AS events can be observed among circadian-regulated homologs within the same species. In the previous study [REF [2] we experimentally validated the majority of novel SJs predicted by RNA-seq for hundreds of randomly selected genes. Among these genes, *CCA1*, a central component of the circadian clock presented an interesting case because its intron 4 retention event was conserved across different plant phyla. Here we show that such conservation can be extended to the homologs within the same species with overlapping but distinct functions such as in the cases of *CCA1/LHY* and *GRP7/GRP8*. This notion further reinforces a hypothesis of the functional significance of UAS.

Finally, we showed that some circadian-associated genes such as *RVE2* employ a strategy of introducing a PTC via a poison cassette exon (PCE) – a conserved UAS event previously shown to be involved in homeostatic regulation of some mammalian SR splicing factors [REF [8]. To our knowledge this is the first example of such an AS event among circadian genes suggesting that the inclusion of a PTC via a PCE event may be a widespread mechanism, regulating in this instance homeostatic expression of a key circadian gene. Importantly, a time course analysis of the accumulation of RVE2 transcripts (Figure 3C) showed that the PCE isoform may not always mirror precisely the accumulation of its full-length protein-coding counterpart and suggests a possibility that the production of the PTC + transcript may undergo a phase shift under specific diurnal conditions. It is possible that similar to the case of some SR splicing factors, RVE2 expression is regulated by coupling of UAS with mRNA decay.

Combined, the approaches described above allowed us to detect novel AS events in circadian genes which otherwise could fall below statistical significance in RNA-seq analysis because of low isoform abundance at specific times of day and/or environmental conditions resulting in insufficient read coverage. A systematic interrogation of the putative novel splicing events allowed us to validate experimentally that AS of key circadian genes in most cases generates isoforms harboring PTCs. Future studies that explicitly demonstrate regulatory role(s) of UAS in clock function are likely to have major biological implications because the circadian clock orchestrates global gene expression in plants.

*Reviewer's comments:* One of main assertions in this study is that most circadian clock genes have alternative splicing forms. Whether this pattern is circadian gene-specific or most plant genes are extensively alternatively spliced is unclear.

*Authors' response:* Please see our response to the similar question from reviewer #1.

*Reviewer's comments:* To rule out noise hypothesis, abundance of unproductive AS among eukaryotes and conservation of alternative splicing events in relative species were mentioned. To this end, the authors should show direct evidences using plants and circadian genes tested.

*Authors' response:* The "stochastic splicing noise” versus regulated unproductive AS hypothesis is mentioned in the Discussion section of our manuscript. Rapidly accumulating evidence [for examples see references [8,18,19] indicates that unproductive alternative splicing (UAS) coupled with NMD is a regulatory gene expression mechanism that is widespread across eukaryotes. Even though our study suggests that UAS could be an important regulatory mechanism for several circadian genes it is not designed to directly resolve this overarching biological question. The references with the examples supporting "conservation of AS events between different species" have been added to the Discussion section.

Reviewer 3: Dr. Marcelo Yanovsky

*Reviewer's comments:* Recent work has provided evidence of widespread occurrence of alternative splicing (AS) in plants. Interestingly, genetic approaches have revealed an important role for AS in the proper regulation of circadian rhythms in plants. The manuscript by Filichkin and Mockler contributes to expand our knowledge of the interplay between AS and circadian networks through a thorough evaluation of AS of plant circadian clock genes using RNA-seq. The authors found that many circadian clock-associated genes in Arabidopsis thaliana are alternatively spliced. The results from RNA-seq data were confirmed using several approaches including RT-PCR, qRT-PCR and/or Sanger sequencing. Most AS events led to the incorporation of in-frame premature stop codons (PTC) through full or partial intron retention. This was a widespread phenomenon among mRNAs from the CCA1/LHY family of MYB transcription factors. Interestingly, an in-frame PTC also resulted from the inclusion of a "poison cassette exon" in one of the homologues of CCA1 known as REVEILLE 2 (RVE2). Intron retention events in the MYB family of transcription factors associated with the clock also took place in the 3´utr region, suggesting they may contribute to regulate mRNA stability. Interestingly, daily oscillations with different phases were observed for the different isoforms of the RVE2 gene. Finally, the authors confirmed and extended previous analysis of the effect of stress treatments on AS of CCA1, showing that the full length transcripts increases in response to cold treatments, whilst the isoform retaining intron 4 increases in response to heat, and these responses are sustained under prolonged stress conditions. The dependence of relative abundance of certain isoforms of clock associated genes on time of day or stress treatments suggest that AS of these genes may contribute to fine-tuning the regulation of physiological processes by the clock, as well as regulation of the clock in response to environmental signals.

*Reviewer's comments:* The manuscript is well written, clear and concise. The data is interesting and presented in a timely fashion for the field, reinforcing the importance of the interplay between AS and the regulation of circadian networks, through changes in AS of core-clock and clock-output genes.

*Reviewer's comments:* As far as data presentation is concerned, it would have been nice to see as part of the main figures, in addition to read coverage graphs that allow the identification of intron retention or exon skipping events, graphic data on exon/exon junctions, which should allow the identification of alternative donor and/or acceptor sites.

*Authors' response:* The graphical illustrations and criteria for coverage of exon/exon junctions by Illumina reads used for the identification of alternative donor and/or acceptor sites are shown in Supplementary Figure 7 of our previous publication [Ref [2].

*Reviewer's comments:* Finally, in the near future, it will be important to start testing the functional significance of the different AS isoforms of the clock genes, and the role of AS in the proper physiological regulation of the circadian network.

*Authors' response:* Indeed, this is very important point. Unproductive alternative splicing (UAS) in circadian clock genes presents a very special case study because the daily oscillations of transcripts levels require rapid and steep adjustments in mRNA levels. Currently, studies of the role of AS in regulation of the circadian network are under way in many labs worldwide using an array of mutant and transgenic lines.

*Reviewer's comments:* The authors mention at the beginning that “To maximize the sensitivity for minor spliced products and to avoid a possible light/dark-dependent shift in isoform ratios, the sampling was done at the phase of peak expression for each tested genes as shown in Figure 1”. Although this approach may simplify the analysis, I do not think it will maximize the sensitivity for minor spliced products. It is possible for instance that the relative abundance of different isoforms changes throughout the day, as shown by the authors for *RVE2*, and therefore this approach may lead to reduced rather than increased sensitivity.

*Authors' response:* We found that this approach works in most cases but we agree that for some genes under specific conditions the effect could be opposite: the unproductive isoform may exhibit different behavior than its productive counterpart. This is why we conducted a time course with limited points here (i.e. *RVE2*) and employ a full time course strategy in the follow up studies.

*Reviewer's comments:* Indeed, I believe the present description of AS events among plant clock associated genes underestimates the total number of events. For example, additional isoforms of *PRR9* have been reported, besides those associated with retention of intron 3, which were not detected/reported here.

*Authors' response:* We agree and clearly state in the paper that our study is likely to underestimate the total number of AS and UAS events in circadian genes. The Supplemental Figure 3, for example, demonstrates that differential intron expression analysis suggests additional intron retention events under heat stress. Undoubtedly, future studies of abiotic or biotic stress treatments, or specific mutations in splicing-related genes will reveal more novel AS patterns.

*Reviewer's comments:* Finally, in Figure 2B, the authors should add the primer that allows amplification of the fully spliced isoform, in the 3´-5´direction.

*Authors' response:* In Figure 2B the primer pairs used for amplification of the fully spliced isoforms have been added.

## Supplementary Material

Additional file 1 **Figure S1. Illumina read coverage of the (A)*LHY*and (B)*LCL1*transcripts.** GBrowse tracks represent untreated control (WT), cold (+4 °C), heat-treated seedlings (+42 °C) and seedlings of the *upf1-1* NMD-impaired mutant. *Arabidopsis* seedlings were grown on plates and treated as described in the Methods. Analogous intron retention events in the 3’ UTRs are indicated by arrows. The *LCL1* IR7 event is shown by brackets. Click here for file

Additional file 3: **Differential coverage of the TAIR10 annotated introns of circadian genes by Illumina reads of samples of plants subjected to heat stress treatment compared to untreated controls.**The normalized read counts and the Q-values were computed using the GENE-Counter statistical RNA-seq package as described [3]. Click here for file

Additional file 2 Oligonucleotide primer sequences and PCR conditions. Click here for file
