# Supplementary material for: Unproductive alternative splicing and nonsense mRNAs: A widespread phenomenon among plant circadian clock genes
Source: Biol Direct. 2012 Jul 2;7:20. doi: 10.1186/1745-6150-7-20 (PMC3403997; doi:10.1186/1745-6150-7-20)
Supplement: Additional file 1 — Figure S1. Illumina read coverage of the (A)LHYand (B)LCL1transcripts. GBrowse tracks represent untreated control (WT), cold (+4 °C), heat-treated seedlings (+42 °C) and seedlings of the upf1-1 NMD-impaired mutant. Arabidopsis seedlings were grown on plates and treated as described in the Methods. Analogous intron retention events in the 3’ UTRs are indicated by arrows. The LCL1 IR7 event is shown by brackets. [file 1745-6150-7-20-S1.ppt]

## Slide 1
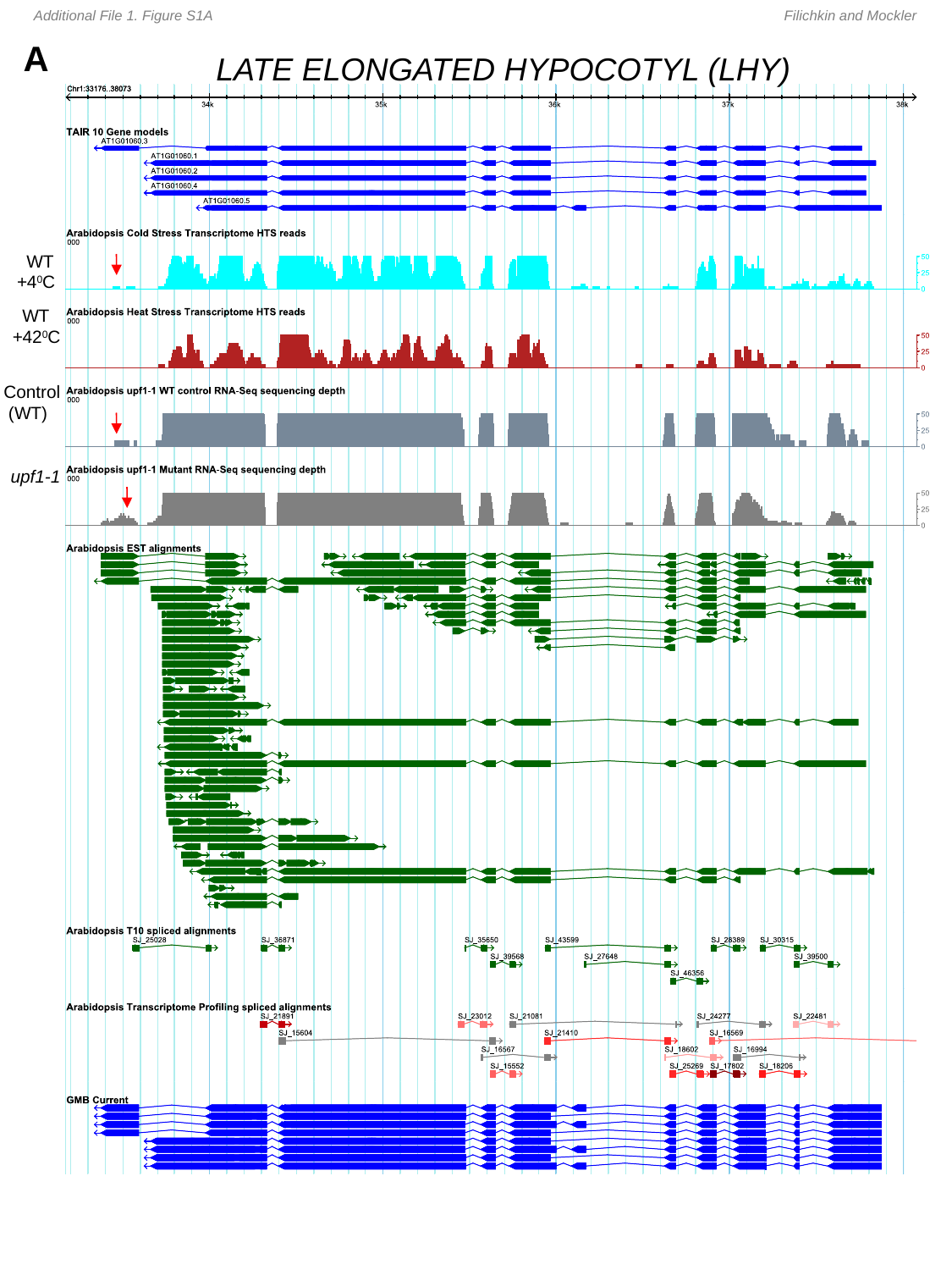

Additional File 1. Figure S1A Filichkin and Mockler
A
LATE ELONGATED HYPOCOTYL (LHY)
 WT
+40C
 WT
+420C
Control
 (WT)
upf1-1

## Slide 2
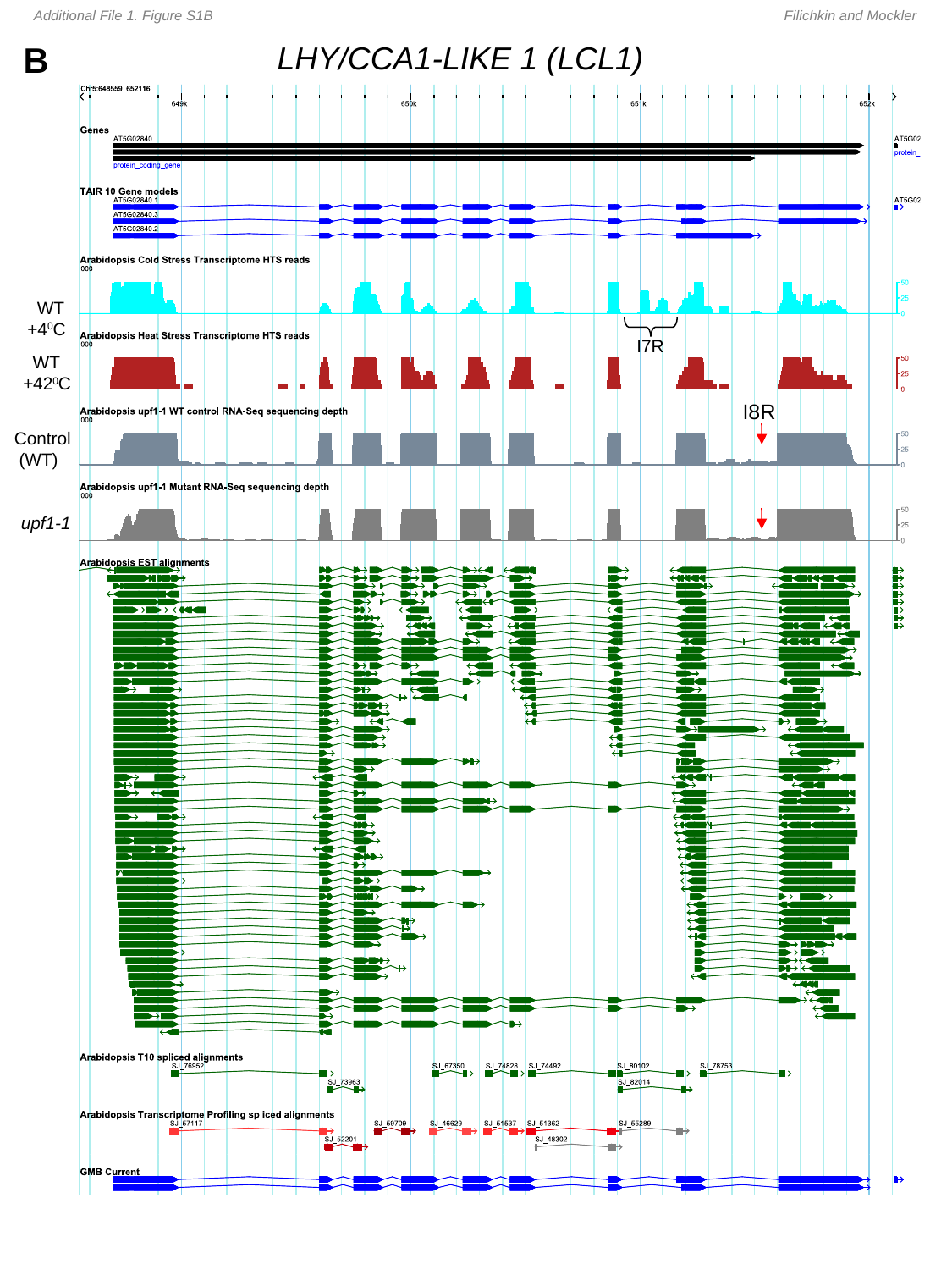

Additional File 1. Figure S1B Filichkin and Mockler
B
LHY/CCA1-LIKE 1 (LCL1)
 WT
+40C
 WT
+420C
Control
 (WT)
upf1-1
I7R
I8R
